# Music stimulates muscles, mind, and feelings in one go

**DOI:** 10.3389/fpsyg.2015.01547

**Published:** 2015-10-08

**Authors:** Stefan Mainka

**Affiliations:** Neurologisches Fachkrankenhaus für Bewegungsstörungen/ParkinsonBeelitz, Germany

**Keywords:** music, gait, training, rhythmic auditory stimulation

Gait and gait related mobility are among the most important factors for the quality of life of patients suffering from Parkinson's disease (PD). In most patients gait and gait related mobility will first lead to patients having to undergo physiotherapy. For gait training in PD there are a variety of evidence-based and well-established therapies. Among such therapies are Nordic walking, treadmill training, dancing, and amplitude training. All of these have proven to be effective in randomized controlled studies (Tomlinson et al., 2013).

One approach that was among the first that had been studied clinically is rhythmic auditory stimulation (RAS). RAS is a therapeutic training technique derived from the concept of neurologic music therapy. In this methodology, music or acoustic stimuli are used and shaped in order to enhance bodily functions such as cognition, speech, or movement (Thaut and Hoemberg, 2014). In RAS rhythmical stimuli or music are used to address and improve gait and gait related issues (Thaut and Rice, 2014).

Music with embedded metronome pulsation has been shown to be most effective in cueing movements. In comparison to single-pulse rhythmic stimulation (like from a metronome) response variability and synchronization offset are markedly reduced. This applies to the frequency range 60–120 bpm, which is relevant for gait training and also other gross and fine motor functions (Thaut et al., 1997). In an experimental study PD patients exhibited spontaneous improvements in gait velocity, cadence, and stride length through the use of functional music with an embedded metronome. A close synchronization between rhythm and step frequency suggested that there was evidence of rhythmic entrainment (McIntosh et al., 1997).

Thaut and colleagues demonstrated in 1996 the effectiveness of a 3-week home based training protocol. Thirty-seven PD patients (at Hoehn and Jahr, 2.5) had been randomly assigned to either RAS with functional training music, self-pacing gait training or no training at all. RAS training consisted of 30 min of daily gait exercises (walking on a flat surface, stair stepping, stop-and-go) with functional music. Music tempo was increased twice within each session by 5–10%. The initial tempo which was derived from the patients normal cadence (100 steps/min = 100 bpm) was also set 5–10% higher every week. The limit for tempo increase was set to 130 bpm. The RAS group improved significantly in velocity (25%) and step cadence (10%) compared with the self-pacing training group (velocity increase of 7%; Thaut et al., 1996).

To this day the work of Thaut and colleagues, in which functional music was used for optimizing gait training in PD patients, has not been reproduced in a randomized and controlled study. Nonetheless in the Cochrane review from 2013 RAS is listed as one of the profoundly evaluated gait training techniques (Tomlinson et al., 2013). This counts mainly for the cueing intervention with metronome, as evidence for the use of music is still scarce.

A second randomized and controlled clinical trial with musically stimulated gait training that was included in the review by Tomlinson et al. reveals profound methodological differences to the RAS technique. In this study by de Bruin, the music was chosen according to patients preference rather than functional criteria. The exercises were not focused on specific therapeutic issues, and most crucially the tempo of music—in Thaut's work the main component of the intervention—varied ±15 bpm from the habitual walking step frequency and was not systematically increased (de Bruin et al., 2010).

It can be stated that there is a lack of sufficient evidence regarding the gait training of PD patients using functionally optimized music. Besides the characteristics of the stimulus (music vs. metronome) the adjustment of tempo in the acoustic stimulation is critical for the therapeutic success.

The optimal stimulation tempo for gait training with RAS is described in several works as 10% above the habitual walking cadence. At this tempo stride length and gait velocity are instantly pushed to an optimal level, while stride-to-stride variability is reduced and dynamic equilibrium is enhanced (Freedland et al., 2002; Willems et al., 2006; Hausdorff et al., 2007; Arias and Cudeiro, 2008, 2010).

Contrarian to this clinical practitioners described that speeding up can lead to a worsened initial heelstrike and provoke a more unsafe walking pattern with smaller steps. This was observed in patients with habitual cadences higher than 114 spm (Mainka and Trebs, 2011). These patients in the clinical praxis of the author often find it difficult to sense and regulate their tempo of steps, and have difficulties positioning feet and torso. In consequence of that accelerated and propulsively declined gait patterns occur. This phenomenon is called festination of gait. The question is whether and how can RAS be applied effectively to this clientele.

Gait festination is described as sharing the same pathological mechanisms as oral festination and freezing of gait (Moreau et al., 2007). This phenomenon is likely to be associated with impaired timing perception, cognitive decline (Riederer and Sian-Hülsmann, 2012) and left-sided symptom dominance (Flasskamp et al., 2012). Thus, for PD patients with a tendency toward festination of gait it might be helpful to address the timing regulation deficit in the RAS training programme. Due to the practical experience of the author these patients benefit from more controlled and measured movements. This can be addressed and facilitated by use of functional music with reduced tempo—namely 95–105 bpm according to the patients habitual cadence (comp. Mainka and Trebs, 2011). This practice is in line with an experiment focused upon PD patients suffering from freezing of gait. Tempo reduction (−10%) through a metronome led to a greater stride length (Willems et al., 2006).

The first clinical study referring to the therapeutic effects of tempo reduction was presented by Benoit in 2014. Fifteen PD patients trained daily for 4 weeks with adjusted functional music. The study was carried out with an uncontrolled and non-randomized one-armed design. The musical tempo was set 10% plus or minus according to the optimal stride length during the initial assessment. Through this paradigm seven subjects practiced RAS with reduced cadence stimulation, while the remaining eight were set to speed up. The whole study sample showed significant improvements in stride length and gait tempo that still persisted in the 1-month-follow-up. Additionally patients also improved in specific perceptive (duration discrimination and beat detection in musical excerpts) and motoric (synchronization with isochronous sequences) timing abilities that had not been trained explicitly (Benoit et al., 2014). Further clinical studies, should highlight the connection between perceptive-cognitive and motor decline and its sensitivity to external tempo regulation.

It has been hypothesized that rhythm and music stimulation might enhance or bypass impaired timing generation by recruiting a cerebellar–thalamic–cortical network. The network also involves the supplementary motor area. This indicates that not only motoric, but also perceptive timing is activated during rhythmic stimulation (Nombela et al., 2013). Music with its integration of time organization (rhythm and meter), attraction of attention (melody, specific sound cues, structure), and emotional activation is likely to play a vital role in these therapeutic issues.

The metronome offers the possibility of a practical and easy tempo control. It is looked at as an objective technical device. This may give the therapist and the patient a sense of functional efficacy and task orientation. Would it be better to stick with the “clean-and-easy” metronome instead of stirring up unwanted feelings during the training?

No, for the majority of the patients it would not be better.

Only music affects human beings beyond timing related mechanisms. There is neuro-anatomical evidence for a dopamine release within healthy individuals as a response associated to the experience of thrills during listening to one's favorite music (Salimpoor et al., 2011). Music instantly stimulates mobility and coordination in PD patients (Bernatzky et al., 2004). The patients are themselves quite clear what best helps them in their walking. 95.5% of 47 patients given the training decided to continue RAS with functional music (German folk style) instead of metronome after having tried both (Mainka, 2014). Music involves mental processing, which can effectively be used for carry over effects (e.g., continuing to hum or sing the melody; Satoh and Kuzuhara, 2008).

There are structural musical criteria for an optimal stimulation of gait training (see Table 1 in Datasheet 1 of Supplementary Material; comp. Thaut et al., 1996). The musical style may be selected according to the patient's preference. This could hold for optimal training motivation and emotional activation. It is noteworthy that the musical genre is exchangeable and essentially independent from structural and physiological functionality. Thus, the music may be rock, pop, folk, country, hip hop, metal, jazz, or another genre as long as structural criteria are incorporated.

The RAS gait training with music should be executed 3–5 times a week for 10–25 min. When appropriate gait maneuvers, stair stepping, stop-and-go exercises, or exercising with walking sticks can be implemented (see Table 2 in Datasheet 2 of Supplementary Material for potential therapeutic goals). With a portable mp3 Walkman it can be carried out anywhere—on the street, on the way to the supermarket, in park or in the countryside. The combination treatment of RAS with Nordic walking or treadmill training may open up additional therapeutic indications, but should be administered carefully with regard to the right stimulation tempo.

It is also important: clinical observations suggest that most patients with atypical Parkinson syndromes do not benefit from RAS. In these diseases (namely progressive supranuclear palsy, multisystemic atrophy, normal pressure hydrocephalus, subcortical arteriosclerotic encephalopathy, cortico-basal degeneration, primary akinesia with gait freezing) impairment of audio-motor entrainment may be related to a more widespread pathology. Thus, before starting RAS therapy it is worth evaluating audio-motor entrainment and considering the likely uncertainty of diagnosis.

Music with its physiological, aesthetic, and emotional qualities may be a highly effective treatment tool for people with Parkinson's disease. Gait, and gait related aspects, can be remedied through training in a joyful and motivating manner, that stimulates muscles, mind and feelings simultaneously.

## Conflict of interest statement

The author declares that the research was conducted in the absence of any commercial or financial relationships that could be construed as a potential conflict of interest.
