# Supplementary material for: Music stimulates muscles, mind, and feelings in one go
Source: Front Psychol. 2015 Oct 8;6:1547. doi: 10.3389/fpsyg.2015.01547 (PMC4597192; doi:10.3389/fpsyg.2015.01547)
Supplement: Supplementary file 1 [file DataSheet1.DOCX]

Table 1. Structural parameter of functional music for gait training

- even meter (2/4 or 4/4)
- stable tempo
- continuous metrically accentuated rhythm
- catchy melody with prominent tones on the beat
- no lyrics
- moderately exalted energy (rhythm & harmony)
- superimposed salient high pitch pulsed metrum
