# Supplementary material for: Music stimulates muscles, mind, and feelings in one go
Source: Front Psychol. 2015 Oct 8;6:1547. doi: 10.3389/fpsyg.2015.01547 (PMC4597192; doi:10.3389/fpsyg.2015.01547)
Supplement: Supplementary file 2 [file DataSheet2.DOCX]

*Table 2. Potential therapeutic goals for RAS gait training with music*

Improvement of:

- stride length
- arm swing
- gait velocity
- walking endurance
- step and trunk symmetry
- upright posture during walking
- initial heelstrike and unrolling of sole of foot
- feeling of security when walking (reduction of anxiety)
- initiation of gait
- tempo regulation
